# Origin of the Rarely Reported High Performance of Mn‐doped Carbon‐based Oxygen Reduction Catalysts

**DOI:** 10.1002/cssc.202200795

**Published:** 2022-08-04

**Authors:** Nagaprasad Reddy Samala, Ilya Grinberg

**Affiliations:** ^1^ Department of Chemistry Bar-Ilan University Ramat Gan Israel 52900

**Keywords:** computational studies, electrocatalysis, metal catalysts, noble-metal free, transition metals

## Abstract

Recent efforts to develop durable high‐performance platinum‐group metal (PGM)‐free oxygen reduction reaction (ORR) electrocatalysts have focused on Fe‐ and Co‐based molecular and pyrolyzed catalysts. While Mn‐based catalysts have advantages of lower toxicity and higher durability, their activity has been generally poor. Nevertheless, several examples of high‐performance Mn‐based catalysts have been reported. Thus, it is necessary to understand why Mn‐based materials much more rarely show high catalytic ORR performance and to determine the factors that can lead to the achievement of such high performance in these rare cases. We have studied the effects of the changes in the macrocycle structure, axial ligand, distance between the active sites, interactions with the dopant N atoms and the presence of an extended carbon network on the ORR catalysis of various Mn‐, Fe‐, and Co‐based systems through the comparison of the adsorption energies of the ORR intermediates. We find that the sensitivity to the local environment changes is the largest for Mn and is the smallest for Co, with Fe between Mn and Co. Our results showed that the strong binding of OH by Mn and the strong sensitivity of the Mn to the modification of its environment necessitate a precise combination of local environment changes to achieve a high onset potential (*V*_onset_) in Mn‐based catalysts. By contrast, the weaker binding of OH by Fe and Co and their weaker sensitivity to local environment changes lead to a wide variety of local environments with favorable catalytic activity (*V*_onset_ *>*0.7 V) for Co‐ and Fe‐based systems. This explains the scarcity of reported Mn‐based pyrolyzed catalysts and suggests that precise material synthesis and engineering of the active site can achieve high‐performance Mn‐based ORR electrocatalysts with high activity and durability.

## Graphical abstract

**Possibility towards ORR**: Here we show the probabilities of the Mn, Fe and Co‐bases catalysts towards ORR. Our results showed that a wide variety of local environments result in favorable catalytic activity for the Co‐ and Fe‐based systems, while for the Mn‐based catalysts a precise combination of local environment changes is necessary to achieve a high onset potential.

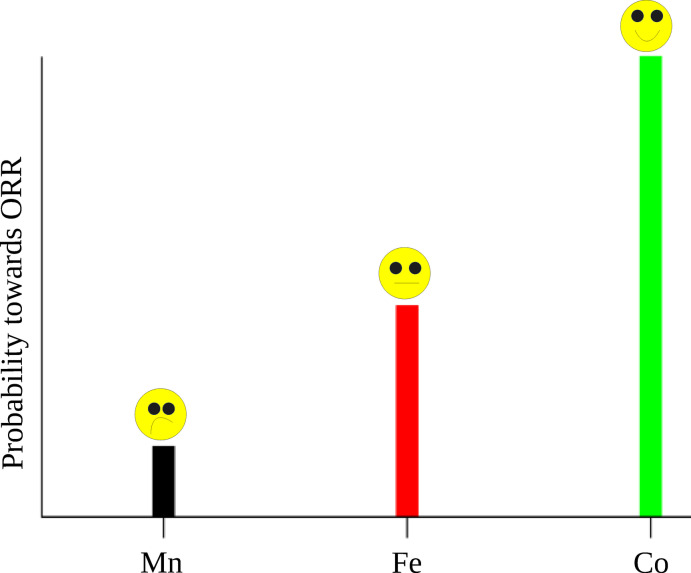

## Introduction

Catalysts play a key role in many industrial processes and their improvement is crucial for the development of the sustainable economy. Catalyst development has been carried out both by trial and error and by rational design. However, due to the increasing energy consumption that gives rise to global environmental issues, accelerated rational design of catalysts for efficient and cost‐effective energy conversion and storage is now urgently necessary. Many of the current catalysts used in energy conversion and storage processes are precious Pt‐group metals (PGM), and their high cost and scarcity severely restrict the use of renewable energy technologies.[^1^, ^2^] This has motivated research into alternative energy conversion catalysts such as non‐precious metal catalysts, single‐atom catalysts and metal‐free catalysts.[^3^, ^4^, ^5^, ^6^, ^7^, ^8^, ^9^]

Over the last decades, intense efforts have been directed at the development of PGM‐free catalysts for the oxygen reduction reaction (ORR) that plays a key role in the electrochemical oxidation of hydrogen in polyelectrolyte membrane fuel cells (PEMFC).[^10^, ^11^] For practical PEMFCs, efficient catalysts are necessary to accelerate the sluggish ORR kinetics in acidic conditions. Furthermore, these catalysts must be able to operate for a long time in harsh PEMFC conditions. Currently, only Pt‐based catalysts satisfy both the efficiency and (to some extent) the durability requirements.[^12^, ^13^] Very recent studies on metal and N‐codoped graphene/carbon nanostructures showed great promise that these materials can replace Pt in fuel cell ORR catalysis.[^10^, ^14^] Various experimental and theoretical studies have been performed on PGM‐free nanostructure with Mn, Fe, Co, Ni, and Cu metal centers, and the observed activities have been found to be comparable to that obtained with the standard Pt catalyst.[^10^, ^14^, ^15^, ^16^, ^17^, ^18^, ^19^, ^20^, ^21^, ^22^, ^23^, ^24^, ^25^] However, the durability of PGM‐free systems is still significantly lower than that of Pt catalysts.[^26^, ^27^] Most studies of PGM‐free ORR catalysts have focused on Fe‐ and Co‐based nitrogen‐doped carbons obtained by pyrolysis from molecular precursors.[^11^, ^28^] In these materials, active sites consist of Fe or Co coordinated by N atoms embedded in the extended carbon framework. While Co‐ and Fe‐based pyrolyzed ORR catalysts are relatively easy to obtain, the presence of Co and Fe has some drawbacks. Co is a toxic metal while the leaching of Fe in the harsh acid PEFMC environment gives rise to the Fenton reaction that promotes the oxidation and degradation of the carbon support.[^29^, ^30^, ^31^] By contrast, Mn is non‐toxic and is not a strong Fenton reagent, so that Mn‐based pyrolyzed catalysts are advantageous compared to Fe‐ and Co‐based materials.^[10]^ Additionally, Mn is more strongly coordinated than Fe and Co, so that Mn‐based catalysts are likely to be more durable. However, to date only a few Mn‐based ORR catalysts that show high performance in acidic conditions have been reported, in contrast to hundreds of studies of Fe‐ and Co‐based ORR catalysts.^[28]^ Thus, it is necessary to understand why Mn‐based materials much more rarely show high catalytic ORR performance and to determine the factors that can lead to the achievement of such high performance in these rare cases.

**Figure 1:**
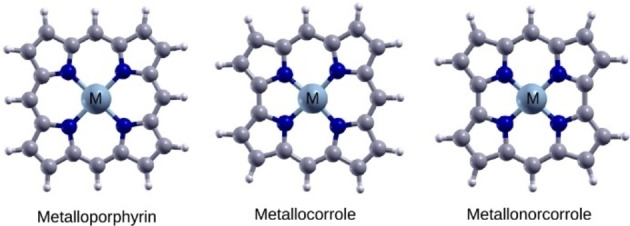
Metalloporphyrin, metallocorrole, and metallonorcorrole structures used in the present study. Cyan, blue, gray and white spheres denote M, N, C and H, respectively.

**Figure 2:**
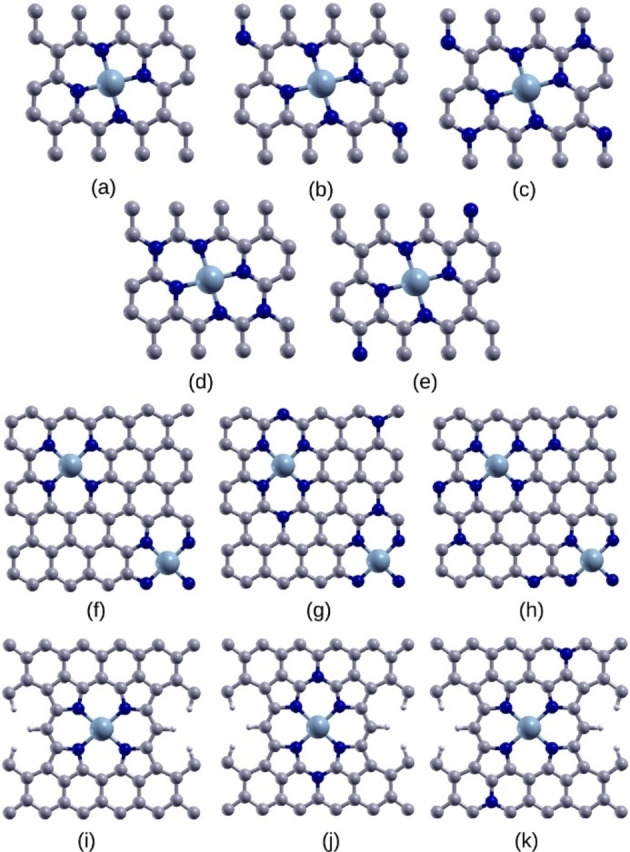
M−N doped graphene and N‐codoped graphene with periodic boundary conditions in the *x*‐ and *y*‐directions. The structures with the small carbon network are labeled as: (a) M−N−C, (b) M−N−C‐2N, (c) M−N−C‐4N, (d) M−N−C‐2N1, and (e) M−N−C‐2N2. The structures with the large carbon network are labeled as: (f) M−N−C−C, (g) M−N−C−C‐2N1, (h) M−N−C−C‐2N2. In addition, the structures from Ref. 10 were also investigated and are labeled as:(i) M−N−C−C‐ref, (g) M−N−C−C‐ref2N1, (h) M−N−C−C‐ref2N2. Cyan, blue, gray and white spheres denote M, N, C and H, respectively.

In this work, we use Mn‐, Fe‐ and Co‐based metalloporphyrins, metallocorroles and metallonorcorroles molecular catalysts (Figure 1) and M−N (M=Mn, Fe and Co) doped and N co‐doped graphene structures (Figure 2) as model ORR catalytic systems and perform first‐principles calculations of ORR energetics to elucidate the roles of structural and chemical motifs governing the ORR activity. A number of factors can affect the reactions at the active sites of PGM‐free electrocatalysts. For example, for molecular catalysts, the identity of the metal center, the structure of the inner ring of the macro‐cycle, axial ligands and substituent groups can all affect the ORR energetics.[^7^, ^32^, ^33^, ^34^] Additionally, the solvation environment such as the pH and the hydrophobic nature of the substrate carbon have been shown to be able to change the ORR pathway.[^35^, ^36^] For pyrolyzed catalysts, in addition to these effects, the spacing between the metal sites and the presence of extended graphitic network can also affect ORR catalysis. Comparison of metalloporphyrins, metallocorroles, (obtained by removing of a single bridging carbon from the porphyrin framework), and metallonorcorroles (obtained by removing two opposite mesocarbons from porphyrin) reveals the effect of the structure of the inner ring of the active site and aromaticity on the ORR energetics, while comparison of the molecular systems to the model doped graphene structures with periodic boundary conditions reveals the effect of the extended carbon network and the effect of the difference between the pyrrole N atoms of the molecular catalyst to the pyridinic N atoms of M−N doped graphene. Additionally, we examine the effect of axial ligands of the metal centers that may be present in pyrolyzed catalysts. Using the onset potential (*V*_onset_) evaluated by DFT calculations as a descriptor of activity, we show that precise material synthesis and engineering of the active site is necessary to obtain highly active Mn‐based catalysts. Such catalysts are intrinsically more likely to be more durable than their Fe‐ and Co‐based counterparts.^[10]^

### Methods

In the ORR reaction, H_2_O molecules are produced from the reaction of the O_2_ molecules with the H^+^ in the solution and the e^−^ provided by the electrode. Ideally, O_2_ is reduced by 4H^+^ and 4e^−^ to give two water molecules. Partial reduction of O_2_ by 2H^+^ and 2e^−^ to produce peroxide is also possible but this pathway is less favorable for energy conversion. The desired four‐electron process proceeds through three intermediate steps with a H^+^ and e^−^ added in each step as described by Equations (1)–3:

(1) $${}^{*}+O{}_{2}+H{}^{+}+e{}^{-}\to {}^{*}\mathrm{OOH},$$

(2) $${}^{*}+O{}_{2}+2H{}^{+}+e{}^{-}\to {}^{*}O+H{}_{2}O,$$

(3) $${}^{*}+O{}_{2}+3H{}^{+}+3e{}^{-}\to {}^{*}\mathrm{OH}+H{}_{2}O.$$

The Sabatier principle states that for an ideal catalyst, the interaction between the adsorbate and adsorbent will be neither too strong nor too weak, enabling relatively easy bond formation and breaking during the catalytic process.^[37]^ The basic Sabatier principle arguments and a simple computational hydrogen electrode (CHE) model proposed by Nørskov et al.^[38]^ have been applied extensively in the computational prediction of electrocatalysts.[^7^, ^9^, ^24^, ^25^, ^32^, ^33^, ^39^] In this approach, the reversible hydrogen electrode (RHE) is used as the reference and for a given applied electrode potential (*U*), the energies can be calculated at any stage based on the addition of the −*neU* term, where *n* is the number of electrons present in that intermediate step. An ideal ORR catalyst should exhibit the adsorption free energies of 1.23, 2.46 and 3.69 eV with respect to water for the hydroxyl complex (*OH), oxo complex (*O), and the hydroperoxyl complex (*OOH), respectively. Therefore, we study the energies of these intermediates in order to estimate the onset potential of the ORR reaction.

All of the calculations were carried out using spin‐polarized density functional theory (DFT) and the rigorously complete plane‐wave (PW) basis set as implemented in the Quantum Espresso‐v6.2 (QE) software package.^[40]^ Pseudopotentials from the GBRV database^[41]^ were used to represent the ion cores. The kinetic energy cutoffs for the charge density and wavefunction were set to 300 and 50 Ry, respectively, in all calculations. The Methfessel–Paxton smearing method was used with the parameter of 0.01 Ry, and simultaneous optimization of the geometry and magnetization was carried out using the default convergence threshold for self‐consistency. We used a large supercell with the *x*‐, *y*‐, and *z*‐axis lattice parameters of ≥25 Å for the vacuum thickness of 15 Å in order to avoid the interactions between the periodic images of the small molecular systems, and due to the large supercell size the Brillouin zone was sampled only at the Γ point. For the M−N doped graphene studies, we used periodic boundary conditions in *x*‐, and *y*‐directions, with the 4× *k*‐point mesh, and to avoid interactions between the periodic images, the lattice parameter in the *z*‐direction was set to 17 Å.

## Results and Discussion

**Figure 3:**
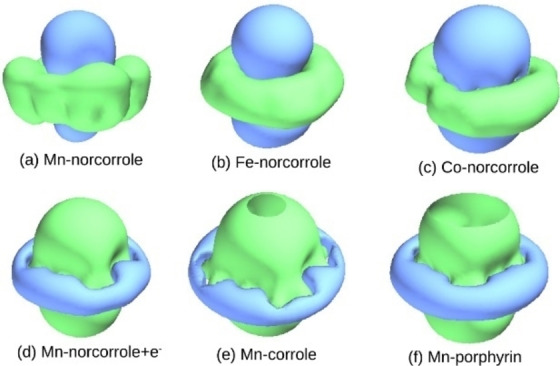
Isochemical shielding surface images of Mn, Fe and Co complexes. Green and blue colors indicate shielding and deshielding regions, respectively.

**Figure 4:**
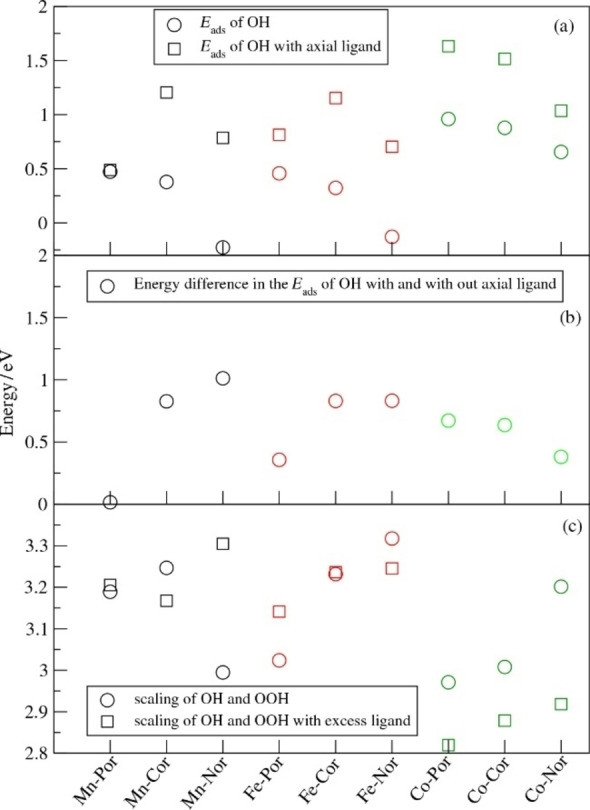
(a) *E*_ads_ of OH with and without axial ligand for the molecular catalysts, (b) the energy difference of *E*_ads,OH_ with and without axial ligand and (c) scaling relations of the Eads values for OH and OOH. Black, red and green symbols correspond to Mn, Fe and Co complexes, respectively.

We first compare the ORR intermediate adsorption energies calculated for the molecular catalysts (Table 1) in order to elucidate the effect of the macrocycle structure and aromaticity on the ORR energetics. We find that macrocycle structure has a strong effect on adsorption energies due to the effect of aromaticity. Removal of first C from porphyrin (Por) to create a corrole (Cor) leads to a slight decrease in the adsorption energies (∼0.1 eV) because of the decreased aromaticity. Then, the removal of the second C to create norcorrole (Nor) leads to much lower energies by (0.2–0.6 eV), due to the change of the macrocycle from aromatic to anti‐aromatic (Figure 3).^[42]^ The addition of the OH/OOH then leads to aromatic behavior and the additional stabilization due to the aromaticity induced by the adsorbed OH/OOH decreases the *E*_ads_ values for Nor systems by 0.8 eV across the board relative to those for Por and Cor (Figure 4a). This effect of decreased *E*_ads_ due to Nor anti‐aromaticity is preserved upon the addition of the axial ligand with the Nor *E*_ads_ values still significantly lower than those for the Por and Cor systems.

**Table 1:** Adsorption energies of ORR intermediates relative to water for metalloporphyrins, metallocorroles and metallonorcorroles in the gas phase, obtained from QE calculations using the complete plane wave basis.

Complex	Adsorption energy [eV]			*V*_onset_
	*OOH	*O	*OH	[V]
Mn‐Por	3.6621	1.2784	0.4730	0.47
H−Mn‐Por	3.6946	2.1168	0.4892	0.49
Mn‐Cor	3.6248	0.9477	0.3781	0.38
H−Mn‐Cor	4.3723	2.4778	1.2048	0.55
Mn‐Nor	2.7677	‐0.1164	‐0.227	–
H−Mn‐Nor	4.0899	1.3478	0.7852	0.78
Fe‐Por	3.4806	1.3266	0.4569	0.46
H−Fe‐Por	3.9553	2.8010	0.8139	0.81
Fe‐Cor	3.5547	1.5781	0.323	0.32
H−Fe‐Cor	4.3893	2.8229	1.1530	0.53
Fe‐Nor	3.1883	0.9567	‐0.1291	–
H−Fe‐Nor	3.9484	2.2433	0.7033	0.70
Co‐Por	3.9308	2.6008	0.9598	0.96
H−Co‐Por	4.4510	3.5572	1.6317	0.47
Co‐Cor	3.886	2.2978	0.8783	0.88
H−Co‐Cor	4.3932	3.4208	1.5146	0.53
Co‐Nor	3.8567	2.0570	0.6552	0.65
H−Co‐Nor	3.9529	2.8025	1.0357	0.97

Comparison of the effect of the axial ligand on the *E*_ads_ of Por, Cor and Nor systems shows that the presence of the axial ligand increases the *E*_ads_ of OOH and OH by 0.2–0.7 eV (Figure 4b). The scaling relation between OOH and OH is preserved, with the differences between the *E*_ads_ values for OOH and OH varying in the 2.8–3.3 eV range. Interestingly, for Mn‐ and Fe‐based systems, the scaling energy difference is in the 3.1–3.3 eV range, while for the Co‐based systems it is in the more favorable 2.8–3.0 eV range (Figure 4c).

**Figure 5:**
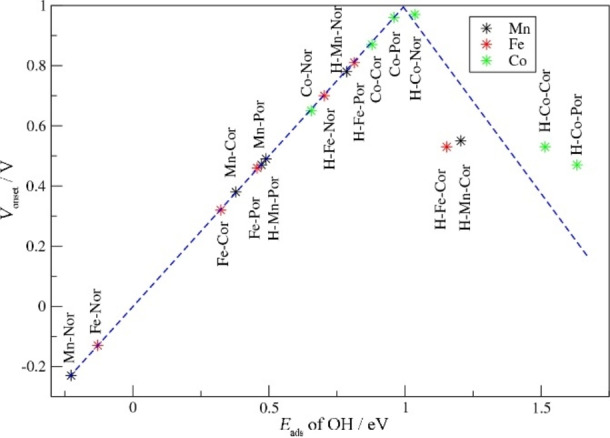
Volcano plot of molecular catalysts obtained from the calculated values of *E*_ads,OH_ and the *V*_onset_ data.

We now discuss the ORR activities of the M−N−C complexes as measured by the onset potential *V*_onset_ (Tables 1, 2, 3). Given the scaling relation between the *E*_ads_ values of OOH and OH, the optimal *V*_onset_ is usually obtained for the *E*_ads_ OH of 0.8–1.0 eV. The strong binding of the Mn and Fe metal centers in the Por, Cor and Nor systems means that the *V*_onset_ values are less than 0.5 eV, making these molecules poor ORR catalysts located to the left side of the volcano curve (Figure 5). The Co‐based Nor also shows strong binding of OH due to the effect of the Nor anti‐aromaticity as described above, so that the *V*_onset_ of Co‐Nor is 0.65 eV. The weaker binding of OH to Co combined with the aromatic Cor and Por frameworks leads to the favorable *V*_onset_ values of 0.87 and 0.96 eV, respectively. As found in our previous study, the addition of the axial ligand to the metal center weakens the O‐metal bond and increases the *E*_ads_ of OH,[^7^, ^32^] shifting the Mn‐ and Fe‐based macrocycles to the right on the volcano curve. In some cases such as for Mn‐Cor, Fe‐Cor, Co‐Cor and Co‐Por, the effect of the axial ligand is too strong, leading to the too weak binding of OH by these macrocycles that overshoots the top of the volcano curve. In this case, OOH adsorption becomes the rate‐determining step. By contrast, for Mn‐Nor, Fe‐Nor and Fe‐Por, the addition of the ligand shifts the system close to the volcano maximum obtaining *V*_onset_ values of 0.78, 0.70 and 0.81 eV, respectively. Interestingly, for the Co‐Nor, the addition of the ligand leads to the *V*_onset_ of 0.97 eV due to the high *E*_ads_ of OH and the difference between the *E*_ads_ values of OOH and OH of only 2.9 eV. This energy difference is slightly more favorable than the usual *E*_ads_ difference of 3.1 eV between OOH and OH obtained for the Mn‐ and Fe‐based systems (Figure 4c).

**Table 2:** Adsorption energies of ORR intermediates relative to water for M−N doped graphene with small carbon network, obtained from QE calculations using the complete plane wave basis.

Complex	Adsorption energy [eV]			*V*_onset_
	*OOH	*O	*OH	[V]
Mn−N‐C	3.5105	0.8877	0.2357	0.23
H−Mn−N‐C	3.8664	2.3002	0.7588	0.76
Mn−N−C‐2N	2.9776	0.8398	0.2447	0.24
H−Mn−N−C‐2N	3.7242	2.1356	0.5914	0.59
Mn−N−C‐4N	2.9025	0.7547	0.1030	0.10
H−Mn−N−C‐4N	3.7144	2.0449	0.5234	0.52
Mn−N−C‐2N1	2.8027	0.7711	0.0206	0.02
H−Mn−N−C‐2N1	3.2896	1.6689	0.2110	0.21
Mn−N−C‐2N2	2.9413	0.8120	0.1599	0.16
H−Mn−N−C‐2N2	3.4847	1.8443	0.4196	0.42
Fe−N‐C	3.4366	1.2592	0.2688	0.27
H−Fe−N−C	4.0414	2.5385	0.9379	0.88
Fe−N−C‐2N	3.4360	1.2716	0.4948	0.49
H−Fe−N−C‐2N	3.7765	2.4572	0.7932	0.79
Fe−N−C‐4N	3.4084	1.7363	0.4750	0.47
H−Fe−N−C‐4N	3.8517	2.2968	0.6648	0.66
Fe−N−C‐2N1	3.4742	1.4248	0.3247	0.32
H−Fe−N−C‐2N1	3.5852	2.2214	0.6035	0.60
Fe−N−C‐2N2	3.4024	1.3363	0.3070	0.31
H−Fe−N−C‐2N2	3.7104	2.3655	0.7429	0.74
Co−N−C	3.7652	2.7032	0.7663	0.77
H−Co−N−C	4.3954	3.5931	1.3557	0.53
Co−N−C‐2N	3.9653	2.8173	0.9401	0.94
H−Co−N−C‐2N	4.0818	3.3582	1.0606	0.84
Co−N−C‐4N	3.9206	2.8628	0.9029	0.90
H−Co−N−C‐4N	3.9979	3.3701	0.9308	0.92
Co−N−C‐2N1	4.1511	2.7164	1.0891	0.77
H−Co−N−C‐2N1	3.6698	3.0518	0.5573	0.56
Co−N−C‐2N2	4.2002	2.7997	1.0496	0.72
H−Co−N−C‐2N2	3.841	3.1511	0.7513	0.75

**Table 3:** Adsorption energies of ORR intermediates relative to water for M−N doped graphene with large carbon network, obtained from QE calculations using the complete plane wave basis.

Complex	Adsorption energy [eV]			*V*_onset_
	*OOH	*O	*OOH	[V]
Mn−N−C−C	3.3735	0.9472	0.2558	0.26
H−Mn−N−C−C	3.8162	2.4132	0.6986	0.70
Fe−N−C−C	3.2744	1.3260	0.2422	0.24
H−Fe−N−C−C	3.7803	2.4090	0.7720	0.77
Co−N−C−C	3.8329	2.7209	0.8229	0.82
H−Co−N−C−C	4.1487	3.4162	1.1435	0.73
Mn−N−C−C‐2N1	3.1094	0.5454	‐0.0521	‐
H−Mn−N−C−C‐2N1	3.3435	1.5198	0.2247	0.22
Mn−N−C−C‐2N2	3.1887	0.6545	0.0380	0.04
H−Mn−N−C−C‐2N2	3.5911	1.8178	0.4929	0.49
Mn−N−C−C‐ref	3.8177	1.7406	0.6466	0.65
H−Mn−N−C−C‐ref	3.8687	2.3016	0.6805	0.68
Mn−N−C−C‐ref2N1	3.7562	1.3568	0.5412	0.54
H−Mn−N−C−C‐ref2N1	3.6218	2.2101	0.5065	0.51
Mn−N−C−C‐ref2N2	3.6910	1.4611	0.5880	0.59
H−Mn−N−C−C‐ref2N2	3.2789	1.7508	0.1404	0.14

We note that while we examined the effect of the H axial ligand here, we can expect this effect to be general to other ligands such as the OH ligand suggested to be present in pyrolyzed catalysts in previous experimental work.^[43]^ Comparison of the OH adsorbate energies for the sample M‐corrole (M=Mn, Fe, Co systems) with H and OH axial ligands (Table S1 in the Supporting Information) found only a small variation (*<*0.2 eV) in the OH adsorption energies for the OH‐ligand system compared to the H ligand system. In particular, the OH ORR intermediate binding energy in the presence of OH ligand attached to Mn differs by only 0.02 eV from the binding energy in the presence of the H ligand as shown in Table S1. Thus, especially for the Mn‐based ORR catalysts, the effects of the OH and H ligands can be expected to be similar, so that the conclusions derived from the H‐ligand calculations are also valid for the systems where OH ligand is more prevalent.

These results show that the manipulation of the structure of the inner ring of the macrocycle in conjunction with the axial ligand is an effective tool for achieving the desired ORR energetics. While for the Co‐based systems, a highly favorable *V*_onset_ of 0.96 eV is already achieved for Co‐Por, and the *V*_onset_ for Co‐Cor is also fairly high at 0.87 eV, the strong binding of OH by the Fe and Mn atoms makes the Fe‐ and Mn‐based systems unsuitable for ORR catalysis. The addition of the axial ligand to Fe‐Por solves this problem, obtaining *V*_onset_ of 0.81 eV. However, the use of Co and Fe in ORR electrocatalysts is unfavorable due to either the toxicity in the original metal state (Co) or to the action of the metal as Fenton reagent in an aqueous systems (Fe).[^29^, ^30^] Therefore, a Mn‐based catalyst is desirable. However, neither the standard Mn‐based Por and Cor (OH binding too strong) nor the Mn‐based Por and Cor with axial ligands (OH binding too weak) show a high *V*_onset_. By contrast, the Mn‐based Nor with the axial ligand achieves the *V*_onset_ of 0.78 that is quite close the values found for the Co‐ and Fe‐based systems. This demonstrates that a combination of several different chemical motifs can be used to transform the previously poorly performing metal centers into high‐performance catalysts.

**Figure 6:**
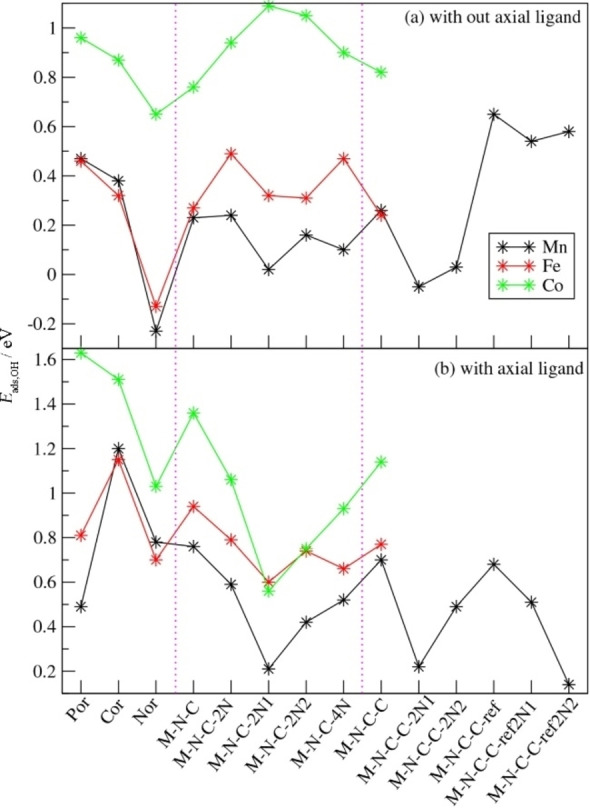
*E*_ads_ data for various molecular, M−N−C and M−N−C−C catalysts with and without axial ligand.

While molecular catalysts are similar to the pyrolyzed and doped graphene catalysts with regard to the local structure of their active site, they lack the extended carbon network and the interactions between the metal and/or nitrogen dopants that are present in bulk carbon catalysts. To elucidate the effects of the extended carbon network and the interactions between active sites, and between active sites and nitrogen dopants, we compare the ORR energetics for several graphene‐based structure with periodic boundary conditions as described above. Here as well, we focus on the OH adsorption energy. Since the carbon framework of the metal‐nitrogen active site environment in the doped graphene structure is similar to that of norcorrole (with the metal participating in two five‐member rings and two six‐member rings in both doped graphene and in the norcorrole), perhaps it can be naively expected that the Eads values of the metal‐N doped graphene structures will be similar to those of the norcorroles. However, an examination of the results (Tables 1 and 3 and Figure 6) shows that the *E*_ads,OH_ values for the M−N−C structures are consistently higher than those of the corresponding norcorroles with increases in *E*_ads_ of 0.12, 0.40 and 0.47 eV for the Co, Fe and Mn M−N−C systems relative to the corresponding norcorroles. This can be understood by considering the effect of aromaticity. While the molecular norcorroles are antiaromatic and this leads to the strong binding of OH by their metal center, the presence of the extended carbon network in the M−N−C and M−N−C−C systems restores aromaticity, leading to the weakened binding of the OH by the metal. However, for all three metals (Co, Fe and Mn), the *E*_ads,OH_ values are still lower than those of the corresponding corroles and porphyrins because the aromatic 4*n*+2π electrons are localized only on the macrocycle in the molecular catalysts.

Comparison of the *E*_ads_ values for small (M−N−C) and large carbon network (M−N−C−C) structures shows that the effect of the change in the distance between the metal active sites varies with the identity of the metal site (Figure 2). While there is no difference in the *E*_ads,OH_ between the small and large Mn−N−C systems, for the Fe−N−C and Co−N−C systems, the *E*_ads,OH_ increases by 0.1–0.25 eV, reaching values similar to those obtained for the corresponding porphyrins. Interestingly, the presence of additional N atom dopants in close proximity to the metal active site has different effects for the three different metals. For Co−N−C, the additional N atoms raise the *E*_ads,OH_ values by 0.2–0.3 eV, while for Fe−N−C−C the increase in the *E*_ads_ is smaller in the 0.05–0.2 eV range and for Mn−N−C−C the additional N atoms actually decrease *E*_ads,OH_ by 0.1–0.25 eV (Figure 6).

In the presence of an axial ligand, the extended carbon network increases *E*_ads_ but with the opposite trend for sensitivity, with Mn showing the weakest effect exerted by the extended carbon network, while the strongest effect of the extended carbon network is observed for Co (Figure 6). The presence of the ligand also reverses the effect of the changes in the metal–metal distance and of additional N dopants, with the increased site‐site distances and additional N dopants actually leading to decreased *E*_ads_ (Figure 6).

Our results have several implications for the pyrolyzed catalysts and doped graphene catalysts widely studied in the literature. First, our results show that by exploiting axial ligands and other variations in the structure, it is possible to achieve a relatively high *V*_onset_ even for the Fe and Mn metals that are located to the left of the peak of the volcano curve for the simple Por and Cor molecular catalysts. For example, the highest *V*_onset_ for the Mn‐based systems is 0.78 eV (for H−Mn‐Nor) while the highest *V*_onset_ for the Fe‐based systems is 0.88 eV (for H−Fe−N−C). Even in the case of Co‐based catalysts for which the Por and Cor systems show high V values, the highest *V*_onset_ of 0.97 eV is obtained for the H−Co‐Nor system, indicating that the presence of an axial ligand and changes in the active site structure can optimize the catalytic activity.

**Figure 7:**
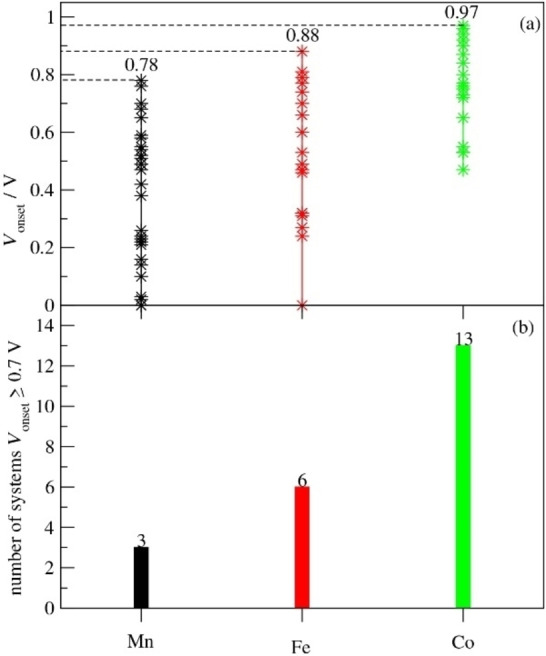
*V*_onset_ data for all Mn, Fe and Co complexes.

Second, consistent with experimental results, the top performance for Co is better than that for Fe which in turn is better than that for Mn. Furthermore, comparing the number of systems with a high *V*_onset_ of 0.70 eV (Figure 7), we see that out of a total of 18 systems (28 in case of Mn) for each metal element, there are three such systems for Mn, six such systems for Fe and 13 such systems for Co. Thus, it is clear that it is much easier to obtain an active site with favorable ORR energetics for a Co‐based system while for an Mn‐based system a “just so” combination of the effects of the metal site structure, axial ligand (or its absence), extended carbon network and co‐dopant N atoms and metal site proximity is necessary to obtain favorable ORR energetics. Fe shows behavior between those of Co and Mn. This is consistent with the experimental results where many Fe‐ and Co‐based high‐performance pyrolyzed ORR catalysts are reported, while only a few such Mn‐based catalysts have been achieved.

This difference between the different metal species can be explained by considering that i) the binding strengths of OH are in order of Mn, Fe, Co so that the Mn Por and Cor and Nor are located strongly to the left of the volcano curve maximum, while Co is close to the volcano curve maximum and Fe is between Co and Mn, and ii) the binding at the Mn active site is more susceptible to the effects of the changes in the active site environment and therefore exhibits greater changes with the modifications of the axial ligand, carbon structure, extended carbon network and additional N dopants. This means that the effects of these factors must be precisely calibrated to shift the Mn‐active site OH binding to the top of the volcano curve rather than undershoot or overshoot the top of the volcano curve. By contrast, the Co active site is already close to the top of the volcano curve for Por and Cor and shows relatively small variations due to the changes in its environment.

Therefore, when MN_4_ (M=Mn, Fe, Co) active sites with a range of different local environments are created by graphene doping or in pyrolyzed catalysts, many of these sites will be active for Co‐based materials, a somewhat smaller fraction of these sites will be active for Fe‐based materials, and only a few sites with highly favorable combination of local environment features will be active for Mn‐based materials. Thus, active Co‐ and Fe‐based materials can be obtained using a wide range of precursors and synthesis conditions, whereas a highly specific combination of local features is necessary to obtain an active Mn‐based ORR catalyst, requiring a particular combination of precursors and synthesis.

Examination of our results (Tables 2 and 3) reveals several design principles for obtaining a high *V*_onset_ in an MnN_4_ system. First, it is necessary to add a ligand to Mn in order to weaken the adsorption of the ORR intermediates and place it closer to the optimal value range. Second, we see that the best performance is obtained for Mn coordinated to pyridinic N, rather than pyrrolic N which binds the adsorbates too strongly. Finally, the presence of graphitic N close to the Mn active site also leads to unfavorably strong binding of the adsorbates and a low *V*_onset_ and should be avoided.

The presence and key role of pyridinic MnN_4_ has been noted in previous experimental work on the MnN_4_‐doped carbon ORR catalysts with high *V*_onset_.^[44]^ Furthermore, experimentally, a high amount of graphitic nitrogen was found in the catalysts, and based on the results obtained in a previous work on FeN_4_ catalysts, it was suggested that these graphitic N atoms play a beneficial role. However, our results suggest that the graphitic N are not beneficial for Mn‐based catalysts, and therefore further improvement in performance can be obtained by decreasing the graphitic N content. Therefore, our work suggests that experimentally, synthesis methods that promote ligation of the active site (e. g., by cross‐linking during synthesis from polymers or through the introduction of the OH ligand^[43]^) should be used. Furthermore, N content should be controlled to decrease the amount of N atoms not involved in the active site in order to avoid the deleterious effects of graphitic N.

We once again note that in contrast to the MnN_4_‐based systems, the CoN_4_‐based systems can show a high *V*_onset_ even without a ligand and in the presence of graphitic N in the vicinity of the active site, while FeN_4_‐based systems require a ligand but are insensitive to the presence of graphitic N. Thus, a wider range of synthesis conditions and method can be used for these systems to achieve high‐performance ORR catalysts.

## Conclusions

We have studied the effects of the changes in the macrocycle structure, axial ligand, distance between active sites, interactions with dopant N atoms and the presence of extended carbon network on the ORR catalysis of various Mn‐, Fe‐, and Co‐based systems by comparing the adsorption energies of the ORR intermediates. We find that aromaticity has a strong effect on the binding of the OH, O and OOH intermediates, with the antiaromatic norcorrole systems showing stronger adsorbate binding than the aromatic porphyrin and corrole systems. The presence of extended carbon network in doped graphene structures for which the metal center has a norcorrole‐like structure restored aromaticity and weakened OH binding to the values slightly lower than those of porphyrins and corroles. The metal‐metal site distance and the presence of additional N atoms also affected the OH binding energies by 0–0.3 eV, moving some systems closer to the volcano curve maximum of the ORR catalytic activity. The sensitivity to the local environment changes was found to be largest for Mn and smallest for Co, with Fe in the middle for systems without an axial ligand, while the opposite was found for the systems with an axial ligand. Overall, our results showed that a wide variety of local environments result in favorable catalytic activity (*V*_onset_ *>*0.7 V) for the Co‐ and Fe‐based systems, while for the Mn‐based catalysts a precise combination of local environment changes is necessary to achieve a high *V*_onset_. This explains the scarcity of reported Mn‐based pyrolyzed catalysts and suggests that further research efforts for precise material synthesis and engineering of the active site can achieve high‐performance Mn‐based ORR electrocatalysts with high activity and durability.

## Conflict of interest

*The authors declare no conflict of interest*.

1

## Supporting information

As a service to our authors and readers, this journal provides supporting information supplied by the authors. Such materials are peer reviewed and may be re‐organized for online delivery, but are not copy‐edited or typeset. Technical support issues arising from supporting information (other than missing files) should be addressed to the authors.

Supporting Information

## Data Availability

The data that support the findings of this study are available from the corresponding author upon reasonable request.
