# Supplementary material for: Origin of the Rarely Reported High Performance of Mn‐doped Carbon‐based Oxygen Reduction Catalysts
Source: ChemSusChem. 2022 Aug 4;15(18):e202200795. doi: 10.1002/cssc.202200795 (PMC9804284; doi:10.1002/cssc.202200795)
Supplement: Supplementary file 1 — Supporting Information [file CSSC-15-0-s001.pdf]

ChemSusChem

Supporting Information

Origin of the Rarely Reported High Performance of Mn-doped Carbon-based Oxygen Reduction Catalysts

Nagaprasad Reddy Samala and Ilya Grinberg*© 2022 The Authors. ChemSusChem published by Wiley-VCH GmbH. This is an open access article under the terms of the Creative Commons Attribution License, which permits use, distribution and reproduction in any medium, provided the original work is properly cited.

Supporting Information

Origin of the Rarely Reported High Performance of Mn-doped Carbon-based Oxygen Reduction Catalysts

Nagaprasad Reddy Samala and Ilya Grinberg *
Department of Chemistry, Bar-Ilan University, Ramat Gan, Israel 52900.

Table of Contents

Item	Description	Pages
Figure S1	Volcano plot of small carbon network systems obtained from E_{ads} of OH and V_{onset} data	S2
Figure S2	Volcano plot of large carbon network systems obtained from E_{ads} of OH and V_{onset} data	S3
Figure S3	V_{onset} data for various molecular, M-N-C and M-N-C-C catalysts with and with out axial ligand.	S4
Table S1	E_{ads} of OH relative to water for metallocorroles with various ligand substitution.	S5
References	References	S6

Iso-chemical shielding surface (ICSS) calculations are carried out by using Gaussian 16[1] and Multiwfn[2,3] software package at B3LYP/6-31+G** level of theory.

Adsorption energies (E_{ads}) of the all the three intermediate steps *OOH, *O and *OH have been calculated with respect to H_2 and H_2O. Here, we first balance the equations of each intermediate step with respect to the final products (*+nH_2O, where n is number water molecules) and then subtract the energies of the reactants from the products as shown in the equations below.

$*OOH+1.5*H_2 \Longrightarrow *+2H_2O$

$*O+H_2 \Longrightarrow *+H_2O$

$*OH+0.5*H_2 \Longrightarrow *+H_2O$

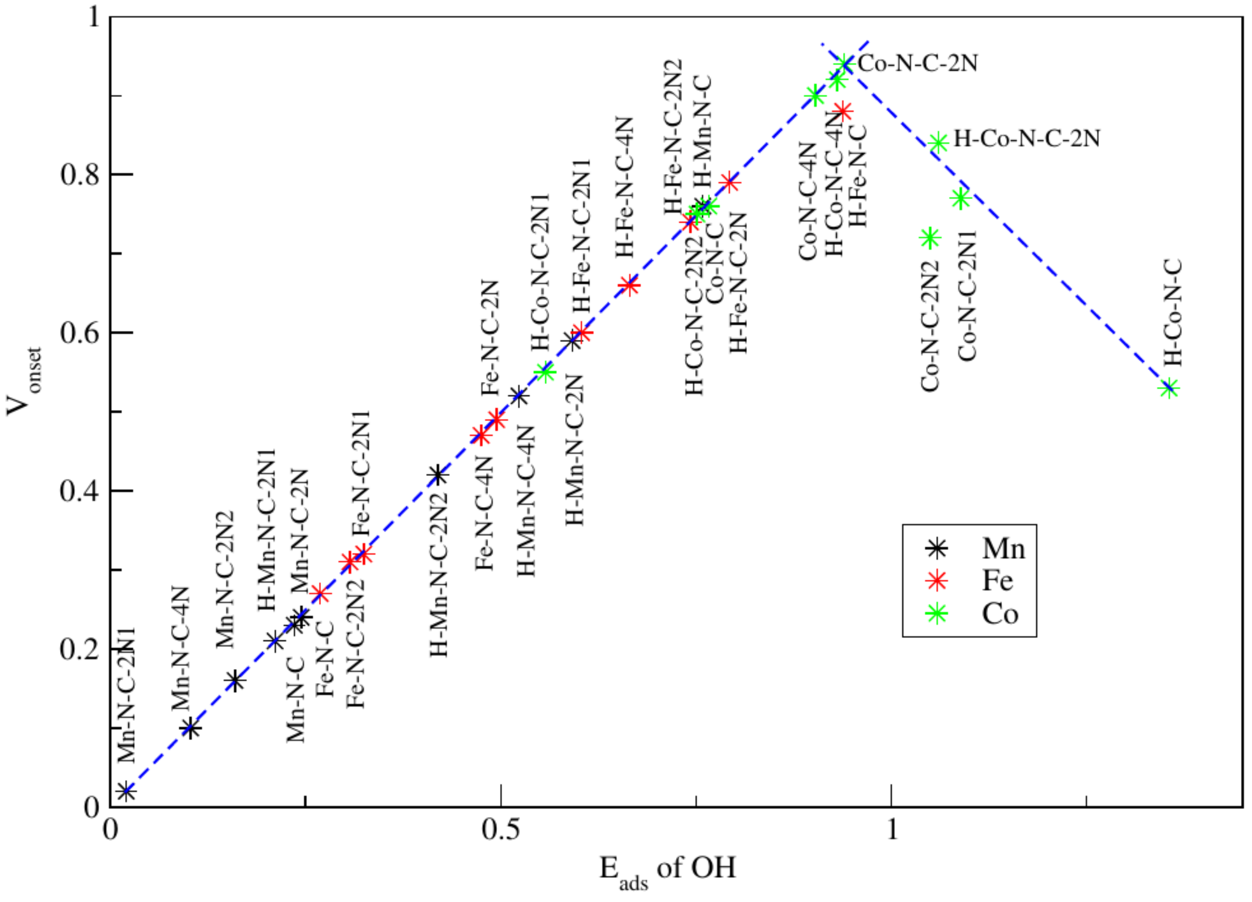

Fig. S1: Volcano plot of small carbon network systems obtained from E_{ads} of OH and V_{onset} data.

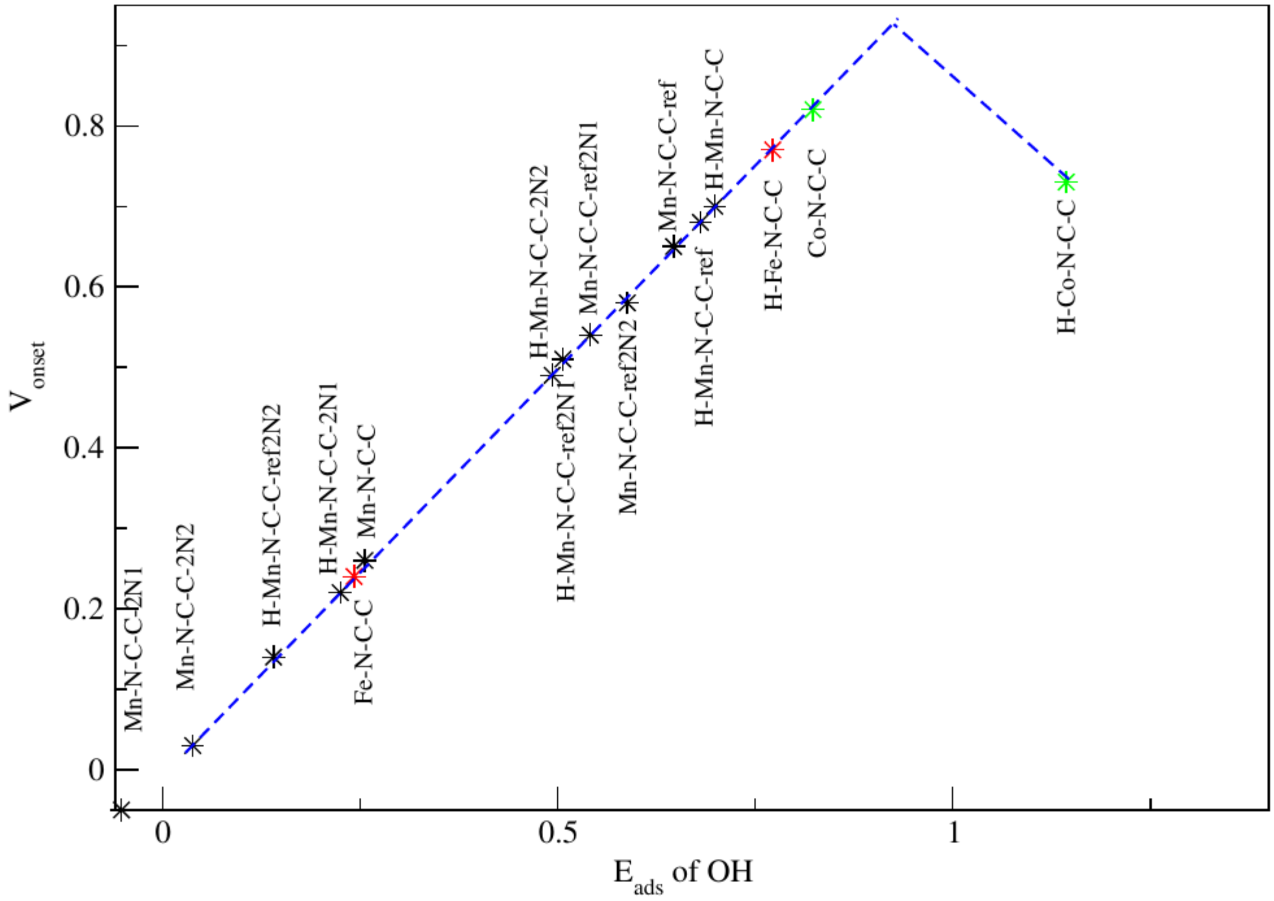

Fig. S2: Volcano plot of large carbon network systems obtained from E_{ads} of OH and V_{onset} data.

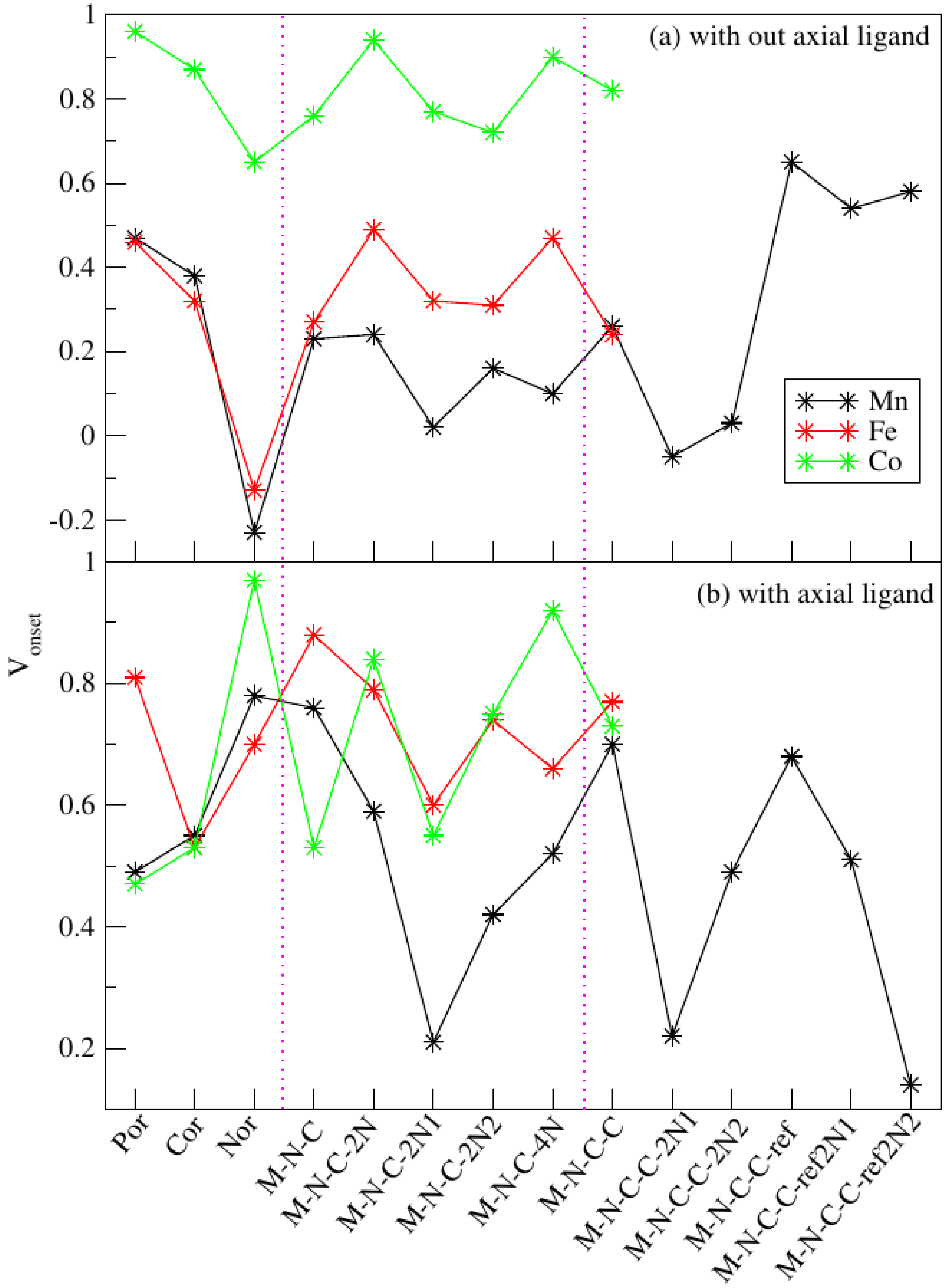

Fig. S3: V_{onset} data for various molecular, M-N-C and M-N-C-C catalysts with and with out axial ligand.

Table S1: E_{ads} of OH relative to water for metallocorroles with various ligand substitution.

Molecule	H	imidazole	CH_3	CF_3	OH
Mn-cor	1.2048	1.1755	1.4301	1.2569	1.2182
Fe-cor	1.1530	1.2456	1.4569	1.2698	1.2291
Co-cor	1.5146	1.2755	1.6102	1.5944	1.2463

References:

1) M. J. Frisch, G. W. Trucks, H. B. Schlegel, G. E. Scuseria, M. A.Robb, J. R. Cheeseman, G. Scalmani, V. Barone, G. A. Peters-son, H. Nakatsuji, X. Li, M. Caricato, A. V. Marenich, J. Bloino, B. G. Janesko, R. Gomperts, B. Mennucci, H. P. Hratchian, J. V. Ortiz, A. F. Izmaylov, J. L. Sonnenberg, D. Williams-Young, F. Ding, F. Lipparini, F. Egidi, J. Goings, B. Peng, A. Petrone, T. Henderson, D. Ranasinghe, V. G. Zakrzewski, J. Gao, N. Rega, G. Zheng, W. Liang, M. Hada, M. Ehara, K. Toyota, R. Fukuda, J. Hasegawa, M. Ishida, T. Nakajima, Y. Honda, O. Kitao, H. Nakai, T. Vreven, K. Throssell, J. A. Montgomery, Jr., J. E. Peralta, F. Ogliaro, M. J. Bearpark, J. J. Heyd, E. N. Brothers, K. N. Kudin, V. N. Staroverov, T. A. Keith, R. Kobayashi, J. Normand, K. Raghavachari, A. P. Ren-dell, J. C. Burant, S. S. Iyengar, J. Tomasi, M. Cossi, J. M. Millam, M. Klene, C. Adamo, R. Cammi, J. W. Ochterski, R. L. Martin, K. Morokuma, O. Farkas, J. B. Foresman and D. J. Fox, *Gaussian 16 Revision A.01*, Gaussian Inc., Wallingford CT, 2016.
2) S. Klod and E. Kleinpeter., *J. Chem. Soc., Perkin Trans.* **2001**, *2*, 1893-1898.
3) T. Lu and F. Chen, *J. Comput. Chem.* **2012**, *33*, 580-592.
